# Quality Control in Eukaryotic Membrane Protein Overproduction

**DOI:** 10.1016/j.jmb.2014.10.012

**Published:** 2014-12-12

**Authors:** Jennifer A. Thomas, Christopher G. Tate

**Affiliations:** MRC Laboratory of Molecular Biology, Cambridge Biomedical Campus, Francis Crick Avenue, Cambridge CB2 0QH, UK

**Keywords:** FSEC, fluorescence-detection size-exclusion chromatography, GPCR, G protein-coupled receptor, HRP, horseradish peroxidase, SEC, size-exclusion chromatography, β_1_AR, β_1_-adrenergic receptor, A_1_R, A_1_ receptor, GFP, green fluorescent protein, PBS, phosphate-buffered saline, eukaryotic membrane proteins, overexpression, G protein-coupled receptors, transporters

## Abstract

The overexpression of authentically folded eukaryotic membrane proteins in milligramme quantities is a fundamental prerequisite for structural studies. One of the most commonly used expression systems for the production of mammalian membrane proteins is the baculovirus expression system in insect cells. However, a detailed analysis by radioligand binding and comparative Western blotting of G protein-coupled receptors and a transporter produced in insect cells showed that a considerable proportion of the expressed protein was misfolded and incapable of ligand binding. In contrast, production of the same membrane proteins in stable inducible mammalian cell lines suggested that the majority was folded correctly. It was noted that detergent solubilisation of the misfolded membrane proteins using either digitonin or dodecylmaltoside was considerably less efficient than using sodium dodecyl sulfate or foscholine-12, whilst these detergents were equally efficient at solubilising correctly folded membrane proteins. This provides a simple and rapid test to suggest whether heterologously expressed mammalian membrane proteins are indeed correctly folded, without requiring radioligand binding assays. This will greatly facilitate the high-throughput production of fully functional membrane proteins for structural studies.

## Highlights

• Discrimination between active and inactive membrane proteins after overexpression is difficult.
• For three G protein-coupled receptors tested in the baculovirus expression system, the vast majority of the protein expressed was inactive.
• Inducible stable mammalian cell lines are far more efficient at producing correctly folded membrane protein.
• A simple assay by Western blotting discriminates active from inactive material.

## Graphical abstract

**Figure:**
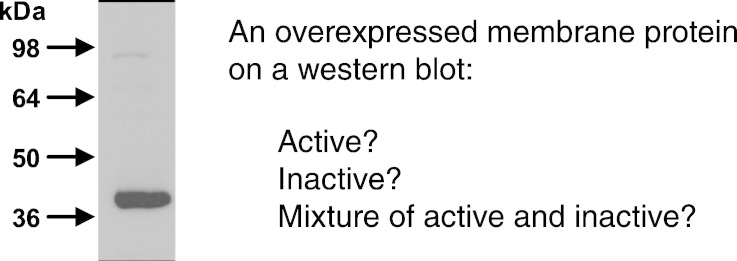

## Introduction

Structure determination of integral membrane proteins requires the production of milligrammes of pure, authentically folded protein for crystallisation [1]. As a natural prerequisite, the protein needs to be expressed in one of a number of heterologous expression systems, such as *Escherichia coli*, yeasts, insect cells or mammalian cells [2]. A number of expression strategies have been developed for each host system and many are now efficient for expression trials of hundreds of proteins in parallel [3]. A popular strategy for the expression of membrane proteins in *E. coli* is to generate fusion proteins with green fluorescent protein (GFP), which can be used as an indicator for both the quantity of protein expressed [4] and its relative stability upon detergent solubilisation by fluorescence-detection size-exclusion chromatography (FSEC) [5]. The utility of this strategy is that fluorescence of the fusion protein expressed in bacteria discriminates between correctly folded membrane protein (the GFP tag is fluorescent) and misfolded, aggregated membrane protein (the GFP tag is not fluorescent) [6], [7]. In *E. coli*, it appears that the misfolded membrane protein promotes the formation of inclusion bodies and, once in an aggregate, the GFP is unable to fold and attain fluorescence. However, in eukaryotic cells, such as yeasts, insect cells used in the baculovirus expression system and in mammalian cells, GFP tagged to a membrane protein remains fluorescent regardless of whether the membrane protein is misfolded in the endoplasmic reticulum or correctly folded in the plasma membrane [8], [9], [10], [11]. Higher eukaryotes have an efficient quality control system in the endoplasmic reticulum so that only folded proteins exit the endoplasmic reticulum, whilst misfolded proteins are retained for degradation [12]. Thus, GFP is not an appropriate marker for the folding status of membrane proteins produced using either mammalian cells or the baculovirus expression system, although it is still useful in analysing the stability of a membrane protein in different detergents by FSEC.

The baculovirus expression system has proven efficient for the production of many eukaryotic membrane proteins, such as G protein-coupled receptors (GPCRs) [13], some of which have been crystallised and their structures determined [14]. However, recombinant baculovirus is not a panacea and there are many proteins that are poorly expressed and there have also been reports that some membrane proteins are expressed predominantly in a misfolded state [2], [15]. This is a serious problem for structural biology, as it is not obvious from current methodology whether an overexpressed membrane protein is predominantly folded or misfolded. If misfolded material is inadvertently purified, then this will likely have a detrimental effect on the ability of the sample to crystallise and may also adversely affect the quality of any crystals obtained. The best way to determine whether misfolded material is present is to perform quantitative Western blotting to assess the total amount of membrane protein expressed in conjunction with radioligand binding assays to determine how much is functional [16], [17]. However, this is expensive, difficult to perform and is also impossible for the majority of membrane proteins that do not possess high-affinity radioligands. It is also unclear whether the presence of misfolded overexpressed membrane protein is a rare event or whether it is commonly observed. We have therefore studied a number of membrane proteins produced both in stable mammalian cell lines and using the baculovirus expression system. The data show that all the four membrane proteins analysed are expressed in the baculovirus system as a mixture of folded and misfolded proteins, whereas mammalian cell lines are much more efficient at producing only correctly folded membrane proteins. A simple comparative detergent solubilisation assay is described, which is an excellent indicator for the presence of misfolded membrane proteins.

## Results

### Comparative expression of the angiotensin II type 1 receptor in insect cells and stable mammalian cell lines

**Fig. 1:**
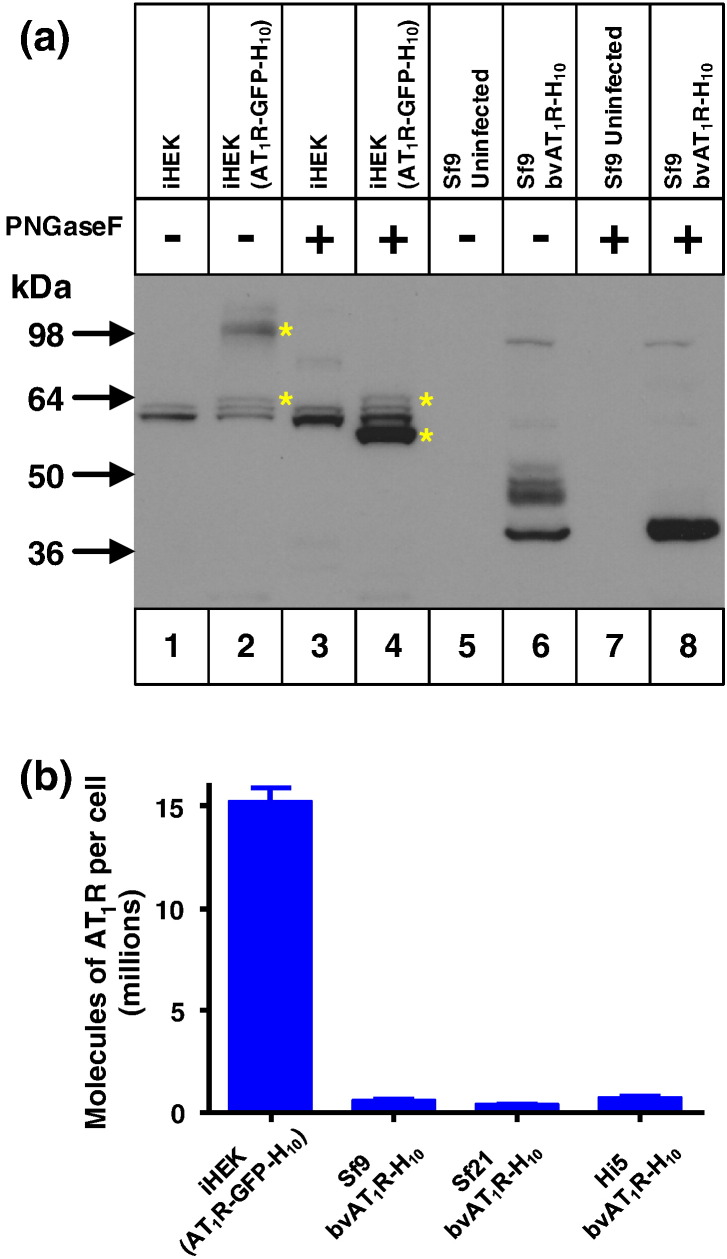
Functional expression of AT_1_R in mammalian cells that is 5-fold higher compared to insect cells. (a) Western blot of whole cells expressing AT_1_R solubilised in SDS. Lanes 1 and 3, iHEK parental cells; lanes 2 and 4, iHEK(AT_1_R-GFP-H_10_) stable clonal cell line; lanes 5 and 7, uninfected Sf9 cells; lanes 6 and 8, bvAT_1_R-H_10_ infected Sf9 cells. N-Linked glycosylation was removed using PNGase F where indicated (+). Bands corresponding to AT_1_R-GFP-H_10_ in mammalian cells are indicated with a yellow asterisk (*). iHEK cell lines were induced with 1 μg/ml tetracycline for 24 h and insect cells were infected with recombinant baculovirus for 48 h. The blot was probed with an anti-pentaHis-HRP conjugated antibody. (b) The amount of functional AT_1_R in each expression system was determined by measuring specific binding of the antagonist [^125^I]Sar^1^. Baculoviral expression was performed in Sf9, Sf21 or Hi5 cells. After the addition of ligand, membranes were solubilised in DDM and non-bound ligand was separated from receptor –ligand complex on gel-filtration spin columns and measured by liquid scintillation counting. [^125^I]Sar^1^-bound AT_1_R is stable in DDM, but not in SDS. The amount of functional AT_1_R was most accurately determined after solubilisation with DDM to ensure that all the receptor was accessible to ligand (see Fig. 2, where twice as much receptor could be measured upon solubilisation in DDM compared to in membranes). Each data point was determined in triplicate from two independent experiments and was plotted as mean ± SEM (*s*tandard *e*rror of the *m*ean).

The human angiotensin II type 1 receptor (AT_1_R) is a GPCR with the typical predicted architecture of seven transmembrane regions with the N-terminus on the extracellular surface of the cell. The receptor contains three N-linked glycosylation sites with one in the N terminal region (Asn4) and two in extracellular loop 2 (Asn176 and Asn188). Two expression systems were used for the production of AT_1_R, the baculovirus expression system and stable tetracycline-inducible mammalian cell lines (the T-Rex system). AT_1_R was expressed with a C-terminal decahistidine tag (H_10_) from the polyhedrin promoter in the recombinant baculovirus bvAT_1_R-H_10_. In inducible mammalian HEK293 cells (iHEK), AT_1_R was expressed with a C-terminal GFP-H_10_ tag from the CMV promoter after induction with tetracycline; the stable cell line iHEK(AT_1_R-GFP-H_10_) was generated through random integration of the plasmid in the genome followed by fluorescence-activated cell sorting to isolate a high-expressing clonal cell line. Initial analysis of expression was performed by Western blotting using an anti-penta-His horseradish peroxidase (HRP) conjugated antibody for detection (Fig. 1). AT_1_R was extensively N-glycosylated in mammalian cells, which could be removed by treatment with PNGase F to yield a major product AT_1_R-GFP-H_10_ of apparent molecular mass of 60 kDa; no unglycosylated AT_1_R-GFP-H_10_ was visible in untreated cells. In Sf9 cells, AT_1_R-H_10_ was expressed as a mixture of glycosylated and unglycosylated receptor, which yielded a single major product (apparent molecular mass of 36 kDa) after treatment with PNGase F. The blot in Fig. 1 contained the same number of cells per lane; thus, an assessment of band intensities by eye suggested that there were similar levels of AT_1_R expressed from the baculovirus expression system and from the stable mammalian cell line. However, despite the apparently similar levels of AT_1_R polypeptide expressed in Sf9 and iHEK cells, radioligand binding assays showed that there was 20× more functional AT_1_R expressed per cell in mammalian cells compared to the best baculoviral expression observed (Fig. 1). This implied that a large proportion of AT_1_R expressed in insect cells was misfolded and incapable of binding antagonist.

**Fig. 2:**
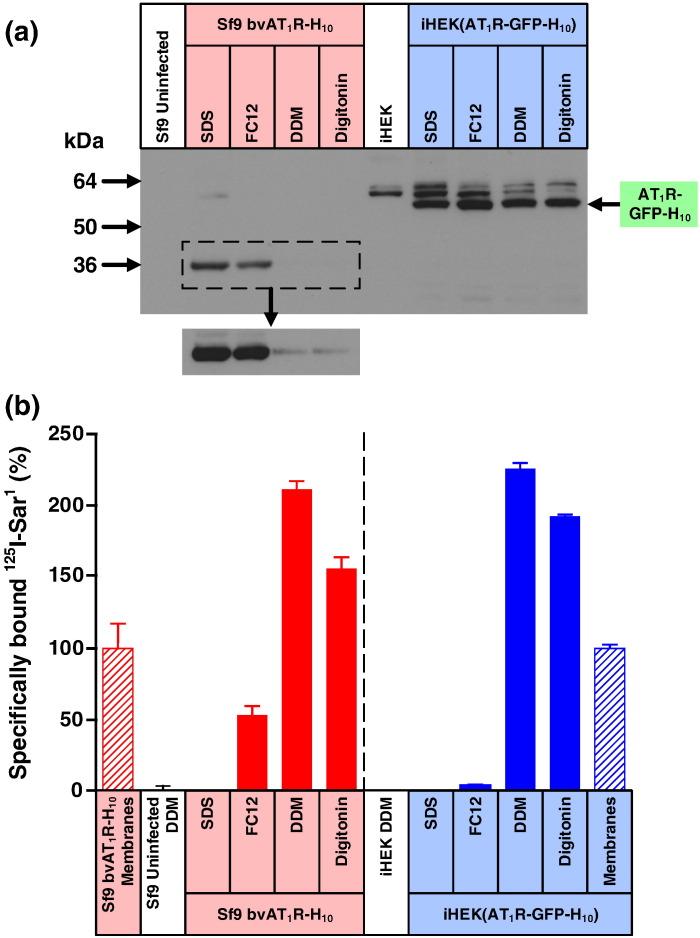
Misfolded AT_1_R produced by the baculovirus expression system is poorly solubilised either by DDM or digitonin. (a) Western blot of AT_1_R solubilised from whole cells using four different detergents (SDS, FC12, DDM or digitonin) and probed with an anti-pentaHis-HRP conjugated antibody. Each lane contains an equal amount of total protein and N-linked glycosylation was removed from all samples using PNGase F prior to SDS-PAGE. AT_1_R was expressed either in the stable mammalian cell line iHEK(AT_1_R-GFP-H_10_) or by using the recombinant baculovirus bvAT_1_R-H_10_ to infect Sf9 cells. The iHEK cell line was induced with 1 μg/ml tetracycline for 24 h and Sf9 cells were infected for 48 h. The Western blot insert is a 7× longer exposure. (b) The amount of functional detergent-solubilised AT_1_R was determined by measuring specific binding of the antagonist [^125^I]Sar^1^. After the addition of ligand, membranes were solubilised in the detergent indicated and non-bound ligand was separated from receptor –ligand complex on gel-filtration spin columns and measured by liquid scintillation counting: red-filled bars, AT_1_R expressed in Sf9 cells; blue-filled bars, AT_1_R expressed in iHEK cells. The amount of AT_1_R in membranes (non-solubilised) was determined by separation of receptor-bound and free radioligand by filtration through glass fibre plates: red hatched bars, AT_1_R expressed in Sf9 cells; blue hatched bars, AT_1_R expressed in iHEK cells. For ease of comparison, binding data have been normalised with respect to AT_1_R in membranes (100%), which is equivalent to 1400 ± 240 dpm (*n* = 2; 380 fmol per million cells) for baculovirus-infected Sf9 cells and 12,000 ± 300 dpm (*n* = 2; 8.8 pmol per million cells) for iHEK(AT_1_R-GFP-H_10_) cells. Absolute levels of AT_1_R therefore cannot be compared meaningfully between the two expression systems using this bar graph (see Fig. 1). Binding assays for AT_1_R contained either 150,000 Sf9 cells or 55,000 iHEK cells. Each data point was determined in duplicate or triplicate from a single experiment and was plotted as mean ± SEM.

Detergent solubilisation is the first step in the purification of a membrane protein; thus, the ability of AT_1_R expressed stably in the iHEK cell line iHEK(AT_1_R-GFP-H_10_) to be solubilised by four different detergents was tested. The four detergents used in order of decreasing “harshness” [18] were sodium dodecyl sulfate (SDS), foscholine-12 (FC12), dodecylmaltoside (DDM) and digitonin. Digitonin was the mildest detergent used and it is very effective in maintaining membrane proteins in a functional state. DDM is one of the most popular mild detergents used for membrane protein purification, but it is a little harsher than digitonin. Only very few bacterial membrane proteins are sufficiently stable to maintain their integrity in either FC12 or SDS; thus, no ligand binding would be expected for AT_1_R solubilised in either SDS or FC12. All four detergents were equally effective at solubilising AT_1_R polypeptide expressed in iHEK cells (Fig. 2). However, as expected from the differing “harshness” of the detergents, only DDM and digitonin maintained the integrity of ^125^I-Sar^1^-bound AT_1_R so that receptor-bound radioligand could be detected (Fig. 2). In contrast, bound radioligand was not detected when SDS was used to solubilise ^125^I-Sar^1^-bound AT_1_R, and only a small amount was detected when FC12 was used. Assays on DDM-solubilised AT_1_R measured nearly twice as much receptor as detected in membranes (Fig. 2), which could be due to freeze–thawed membranes being a mixture of both rightside-out vesicles and inside-out vesicles, and the membrane-impermeant peptide ^125^I-Sar^1^ could bind only to AT_1_R in the rightside-out vesicles.

Detergent solubilisation of ^125^I-Sar^1^-bound AT_1_R from Sf9 cell membranes after expression from the recombinant baculovirus bvAT_1_R-H_10_ followed a similar pattern to that observed from the stable mammalian iHEK cell line; that is, double the amount of radioligand was observed in DDM-solubilised receptor compared to membranes and no binding was detected when SDS was used. Note that the binding data in Fig. 2 are normalised for ease of comparison, whereas in actuality, there is 20-fold less functional AT_1_R per cell in Sf9 cells compared to the stable mammalian cell line. However, the Western blotting data of AT_1_R produced in Sf9 cells are different from the analogous data from the iHEK cell line. Orders of magnitude more AT_1_R polypeptide was solubilised from Sf9 cell membranes by SDS or FC12 compared to either DDM or digitonin (Fig. 2). It is reasonable to assume from the ^125^I-Sar^1^ binding data that DDM solubilised all the functional AT_1_R and therefore the difference between the signal on the Western blot for DDM-solubilised AT_1_R and SDS-solubilised AT_1_R represents misfolded AT_1_R.

**Fig. 3:**
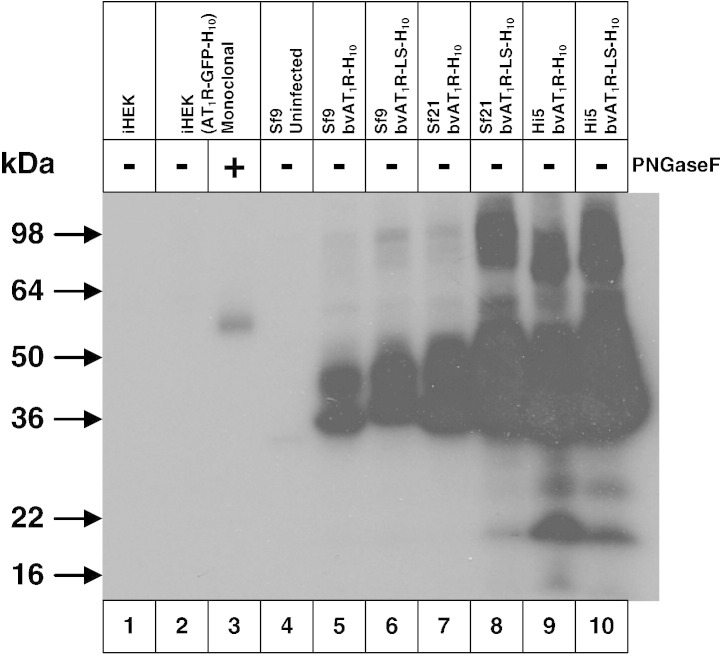
DDM solubilises considerable amounts of inactive AT_1_R produced in the baculovirus expression system. (a) Western blot of DDM-solubilised AT_1_R, with equal amounts of active receptor per sample (lanes 2, 3 and 5–10). The blot was probed with an anti-pentaHis-HRP conjugated antibody. Lane 1, iHEK parental cells; lanes 2 and 3, iHEK(AT_1_R-GFP-H_10_) stable clonal cell line; lane 4, uninfected Sf9 cells; lanes 5–10, bvAT_1_R-H_10_ infected insect cells. N-Linked glycosylation was removed using PNGase F where indicated (+). AT_1_R was expressed either in the stable mammalian cell line iHEK(AT_1_R-GFP-H_10_) or by using the recombinant baculoviruses bvAT_1_R-H_10_ and bvAT_1_R-LS-H_10_ to infect Sf9, Sf21 and Hi5 cells as indicated. iHEK cell lines were induced with 1 μg/ml tetracycline for 24 h and insect cells were infected with recombinant baculovirus for 48 h. The amount of functional AT_1_R was determined by measuring specific binding of the antagonist [^125^I]Sar^1^.

There is a significant difference between the Western blotting signal for SDS-solubilised AT_1_R and DDM-solubilised AT_1_R from Sf9 cells and that difference represents an amount of misfolded AT_1_R that can be solubilised by SDS but not by DDM. However, there may be more misfolded AT_1_R present in Sf9 cell membranes than suggested from the differential solubility in SDS *versus* DDM because it is plausible that DDM can also solubilise some AT_1_R that cannot bind antagonist. To assess this possibility, we diluted membranes from the stable mammalian cell line iHEK(AT_1_R-GFP-H_10_) and insect cell membranes expressing AT_1_R-H_10_ to give the same amount of functional AT_1_R per millilitre, solubilised in DDM and then analysed by Western blotting. The data (Fig. 3) showed clearly that there was considerably more AT_1_R polypeptide solubilised from Sf9 cells than from the mammalian cell line and that this difference is due to misfolded receptor given that there was the same amount of functional receptor per lane. Efforts to decrease the amount of misfolded AT_1_R in the baculovirus expression system either by using different cell lines (Sf21, Hi5) or by including an N-terminal signal sequence on AT_1_R were ineffective (Fig. 3).

**Fig. 4:**
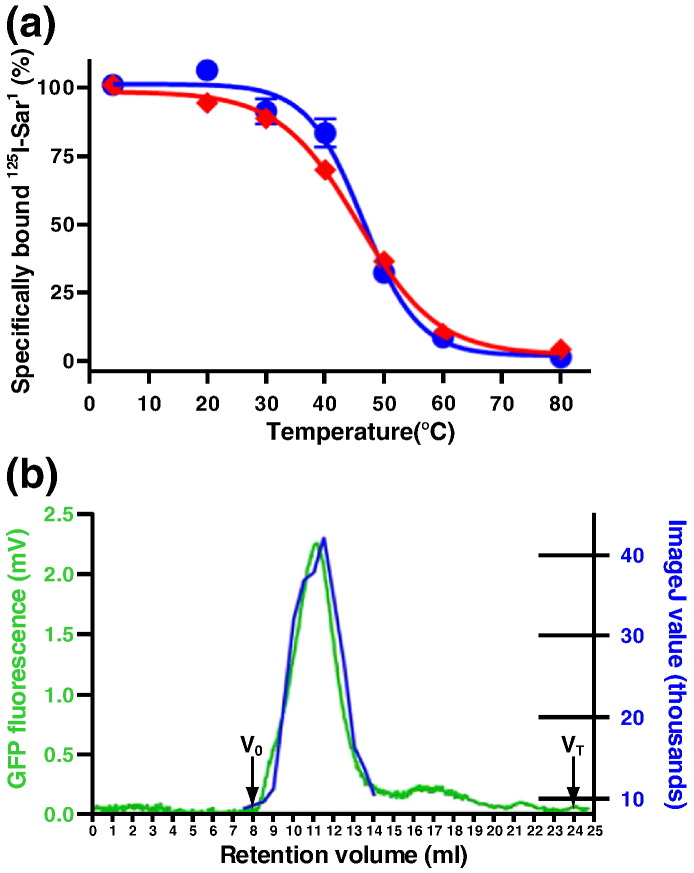
Comparison of AT_1_R expressed in mammalian cells and insect cells. (a) Stability of DDM-solubilised AT_1_R bound to the antagonist [^125^I]Sar^1^. AT_1_R was expressed using three different expression systems: blue circles, baculovirus bvAT_1_R in Sf9 cells; red diamonds, stable clonal cell line iHEK(AT_1_R-GFP-H_10_). The apparent *T*_m_ values of AT_1_R expressed in each system are as follows: Sf9 cells, 46 ± 0.8 °C; iHEK(AT_1_R-GFP-H_10_), 46 ± 0.7 °C. Each data point was determined in triplicate and was plotted as a mean value ± SEM. (b) SEC was carried out using a Superdex 200 10/300 (24 ml) column. The elution of iHEK(AT_1_R-GFP-H_10_) was detected using GFP fluorescence (mV). The elution of bvAT_1_R-H_10_ was detected by Western blotting and band quantification (ImageJ value). iHEK(AT_1_R-GFP-H_10_) shows a symmetrical peak whereas bvAT_1_R-H_10_ shows two peaks; however, both systems show elution of a protein of a similar size. The void (*V*_o_) and total (*V*_T_) column volumes are indicated.

In order to ascertain the quality of AT_1_R expressed in either mammalian cells or insect cells, we analysed two biophysical parameters of the detergent-solubilised receptor. Firstly, the thermostability of DDM-solubilised AT_1_R was determined and the apparent *T*_m_ values of ^125^I-Sar^1^-bound AT_1_R expressed in either Sf9 cells or mammalian cells were found to be identical (Sf9 cells, 46 ± 0.8 °C; iHEK(AT_1_R-GFP-H_10_), 46 ± 0.7 °C). Secondly, the mobility on size-exclusion chromatography (SEC) of AT_1_R expressed using the baculovirus expression system in Sf9 cells or from the stable mammalian cell line was compared and also found to be very similar (Fig. 4). These data, coupled with the similarity in pharmacology between AT_1_R expressed in the two cell types [19], suggest that there is no significant difference between correctly folded AT_1_R produced in the baculovirus expression system and AT_1_R produced in the stable mammalian cell line.

### The presence of misfolded protein upon overexpression from recombinant baculovirus is not uncommon

**Fig. 5:**
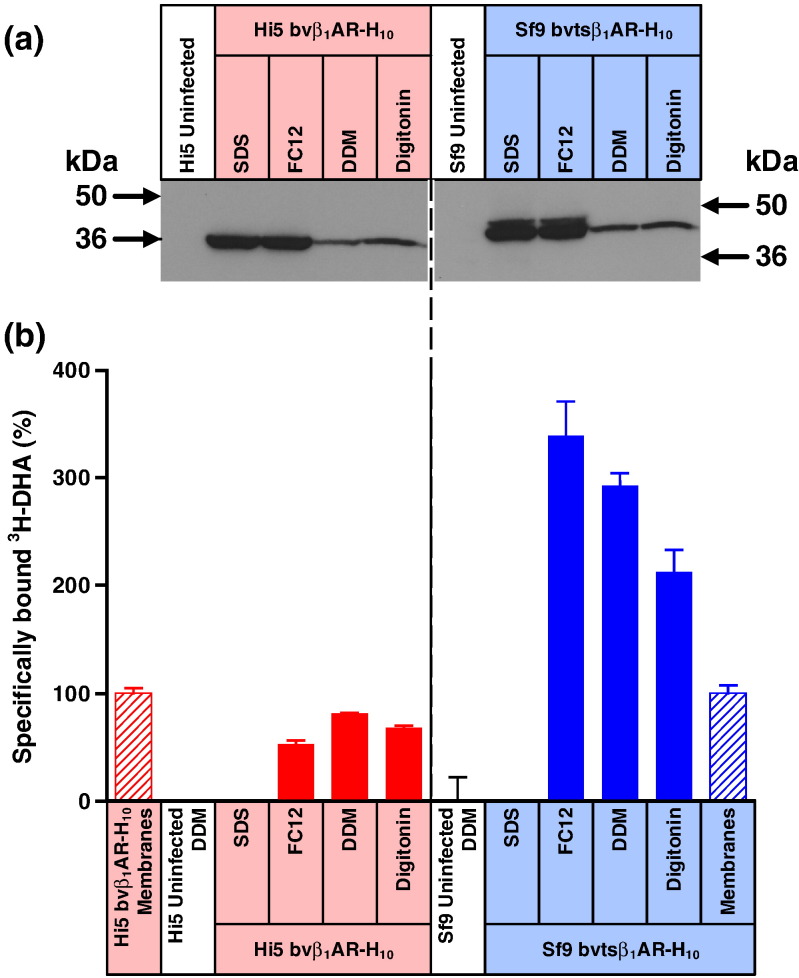
Misfolded β_1_AR is poorly solubilised either by DDM or digitonin. (a) Western blot of β_1_AR solubilised from whole cells using four different detergents (SDS, FC12, DDM or digitonin) and probed with an anti-pentaHis-HRP conjugated antibody. Each lane contains an equal amount of total protein. β_1_AR was expressed by using the recombinant baculovirus bvβ_1_AR-H_10_ to infect Hi5 cells. tsβ_1_AR was expressed by using the recombinant baculovirus bvtsβ_1_AR-H_10_ to infect Sf9 cells. Hi5 and Sf9 cells were infected for 48 h. The broken line indicates two separate blots. (b) The amount of functional detergent-solubilised β_1_AR and tsβ_1_AR was determined by measuring specific binding of the antagonist [^3^H]DHA. After the addition of ligand, membranes were solubilised in the detergent indicated and non-bound ligand was separated from receptor–ligand complex on gel-filtration spin columns and measured by liquid scintillation counting: red-filled bars, β_1_AR expressed in Hi5 cells; blue-filled bars, tsβ_1_AR expressed in Sf9 cells. The amount of β_1_AR in membranes (non-solubilised) was determined by separation of receptor-bound and free radioligand by filtration through glass fibre plates: red hatched bars, β_1_AR expressed in Hi5 cells; blue hatched bars, tsβ_1_AR expressed in Sf9 cells. For ease of comparison, binding data have been normalised with respect to β_1_AR in membranes (100%), which is equivalent to 11,000 ± 550 dpm (*n* = 3; 6.1 pmol per million cells) for baculovirus-infected Hi5 cells and 2600 ± 190 (*n* = 3; 1.4 pmol/l) for bvtsβ_1_AR-H_10_ infected Sf9 cells. All binding assays for β_1_AR and tsβ_1_AR contained 8300 cells, and therefore, comparison on absolute levels of receptor can be directly compared. Each data point was determined in duplicate or triplicate from a single experiment and was plotted as mean ± SEM.

The presence of substantial amounts of misfolded AT_1_R upon production in the baculovirus expression system raised the question of whether this is specific for AT_1_R or whether other membrane proteins also exhibited this property. As it is not possible to test rigorously all membrane proteins, a careful selection was made of interesting test cases. The avian β_1_-adrenergic receptor (β_1_AR) is a well-characterised GPCR and its structure has been determined bound to many different ligands of different efficacy [20], [21], [22], [23]. All of the β_1_AR crystals were grown from protein expressed in either Sf9 or Hi5 cells using recombinant baculoviruses [24], [25]. The assays described for AT_1_R were therefore repeated using wild-type β_1_AR with truncations at the N-terminus and at the C-terminus (bvβ_1_AR-H_10_), which facilitates expression of a homogenous protein. Comparison of the amount of β_1_AR-H_10_ solubilised by the different detergents clearly indicates that a large proportion of the receptor is indeed misfolded, as suggested by the higher proportion of receptor solubilised by either SDS or FC12 compared to either DDM or digitonin (Fig. 5). Efforts to improve the proportion of folded protein by using a thermostable β_1_AR mutant fused at the N-terminus to a leader sequence and a well-folded soluble protein (thioredoxin) did not increase the proportion of correctly folded β_1_AR (Fig. 5). However, it is interesting to note that receptor containing an uncleaved leader sequence was only extracted by SDS or FC12, suggesting that this sub-population of receptor was probably mainly misfolded. In addition, it is unlikely that the fusion protein was efficiently trafficked to the cell surface given that FC12 extraction resulted in a 3-fold increase in the amount of receptor binding obtained compared to when membranes were used.

**Fig. 6:**
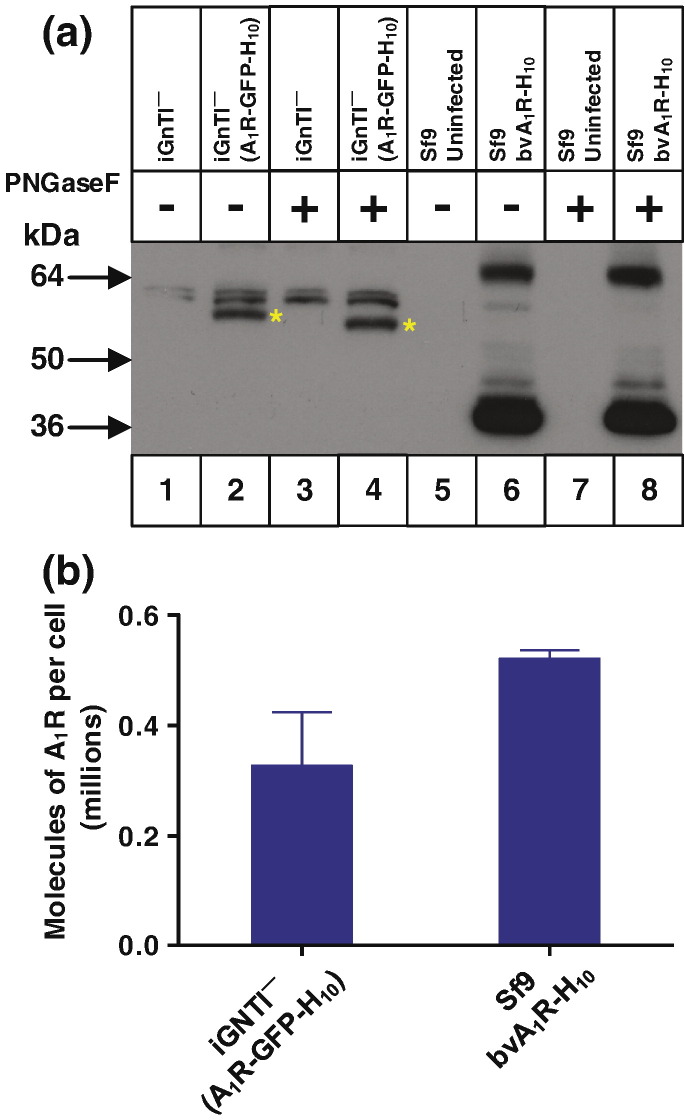
Expression of A_1_R in mammalian cells compared to insect cells. (a) Western blot of whole cells expressing A_1_R solubilised in SDS. Lanes 1 and 3, iGnTI^−^ parental cells; lanes 2 and 4, iGnTI^−^(A_1_R-GFP-H_10_) stable cell line; lanes 5 and 7, uninfected Sf9 cells; lanes 6 and 8, bvA_1_R-H_10_ infected Sf9 cells. N-Linked glycosylation was removed using PNGase F where indicated (+). Bands corresponding to A_1_R-GFP-H_10_ in mammalian cells are indicated with a red asterisk (*). The iGnTI^−^ cell line was induced with 1 μg/ml tetracycline for 24 h and insect cells were infected with recombinant baculovirus for 72 h. The blot was probed with an anti-pentaHis-HRP conjugated antibody. (b) The amount of functional A_1_R in each expression system was determined by measuring specific binding of the antagonist [^3^H]DPCPX. After the addition of ligand, membranes were solubilised in DDM and non-bound ligand was separated from receptor–ligand complex on gel-filtration spin columns and measured by liquid scintillation counting. Each data point was determined in duplicate and was plotted as mean ± SEM.

**Fig. 7:**
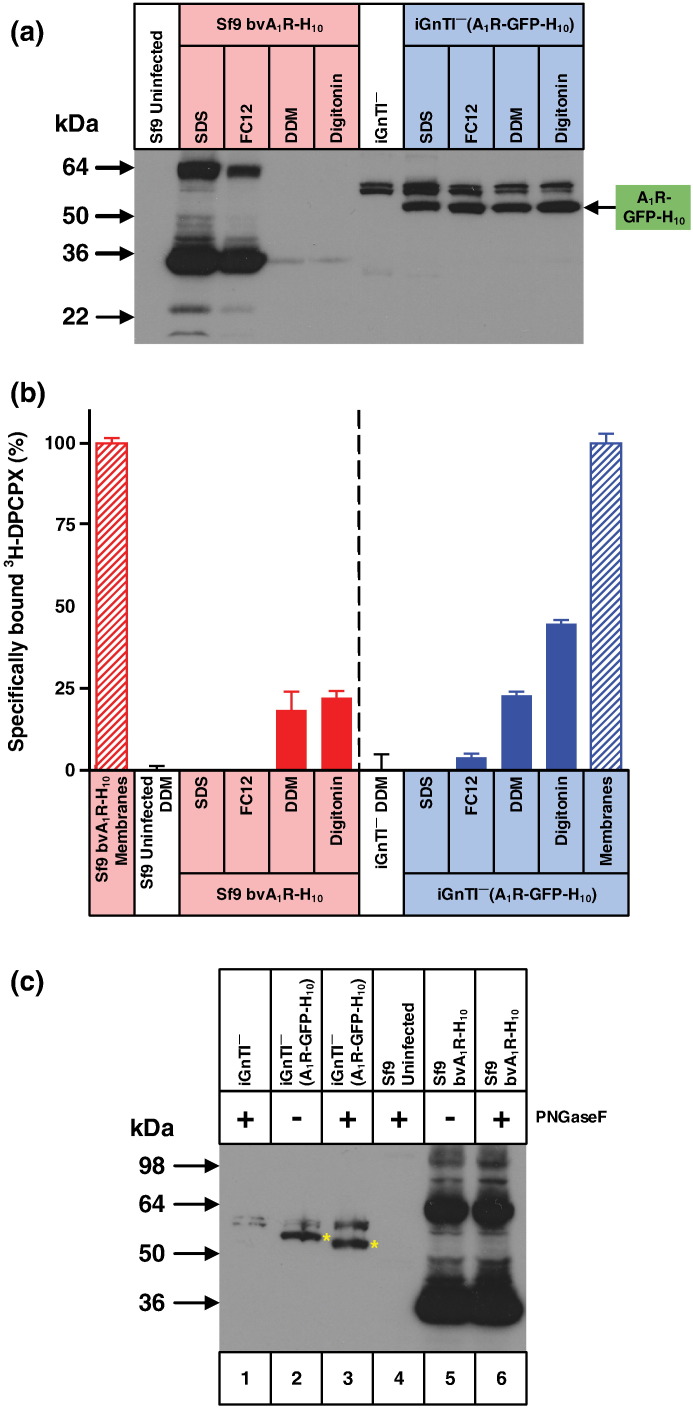
Misfolded A_1_R produced by the baculovirus expression system is poorly solubilised either by DDM or digitonin. (a) Western blot of A_1_R solubilised from whole cells using four different detergents (SDS, FC12, DDM or digitonin) and probed with an anti-pentaHis-HRP conjugated antibody. Each lane contains an equal amount of total protein and N-linked glycosylation was removed from all samples using PNGase F prior to SDS-PAGE. A_1_R was expressed either in the stable mammalian cell line iGnTI^−^(A_1_R-GFP-H_10_) or by using the recombinant baculovirus bvA_1_R-H_10_ to infect Sf9 cells. The iGnTI^−^ cell line was induced with 1 μg/ml tetracycline for 24 h and Sf9 cells were infected for 72 h. (b) The amount of functional detergent-solubilised A_1_R was determined by measuring specific binding of the antagonist [^3^H]DPCPX. After the addition of ligand, membranes were solubilised in the detergent indicated and non-bound ligand was separated from receptor–ligand complex on gel-filtration spin columns and measured by liquid scintillation counting: red-filled bars, A_1_R expressed in Sf9 cells; blue-filled bars, A_1_R expressed in iGnTI^−^ cells. The amount of A_1_R in membranes (non-solubilised) was determined by separation of receptor-bound and free radioligand by filtration through glass fibre plates: red hatched bars, A_1_R expressed in Sf9 cells; blue hatched bars, A_1_R expressed in iGnTI^−^ cells. For ease of comparison, binding data have been normalised with respect to A_1_R in membranes (100%), which is equivalent to 120,000 ± 2000 dpm (*n* = 3; 2.9 pmol per million cells) for baculovirus-infected Sf9 cells and 7500 ± 250 dpm (*n* = 3; 3.8 pmol per million cells) for iGnTI^−^(A_1_R-GFP-H_10_) cells. Absolute levels of A_1_R therefore cannot be compared meaningfully between the two expression systems using this bar graph. Binding assays for A_1_R contained either 150,000 Sf9 cells or 7500 iGnTI^−^ cells. Each data point was determined in duplicate or triplicate from a single experiment and was plotted as mean ± SEM. (c) Western blot of DDM-solubilised A_1_R, with equal amounts of active receptor per sample (lanes 2, 3, 5 and 6). The blot was probed with an anti-pentaHis-HRP conjugated antibody. Lane 1, iGnTI^−^ parental cells; lanes 2 and 3, iGnTI^−^(A_1_R-GFP-H_10_) stable cell line; lane 4, uninfected Sf9 cells; lanes 5 and 6, bvAT_1_R-H_10_ infected Sf9 cells. N-Linked glycosylation was removed using PNGase F where indicated (+). A_1_R was expressed either in the stable mammalian cell line iGnTI^−^(A_1_R-GFP-H_10_) or by using the recombinant baculovirus bvA_1_R-H_10_ to infect Sf9 cells. Bands corresponding to A_1_R-GFP-H_10_ in mammalian cells are indicated with a yellow asterisk (*). The iGnTI^−^ cell line was induced with 1 μg/ml tetracycline for 24 h and insect cells were infected with recombinant baculovirus for 72 h. The amount of functional A_1_R was determined by measuring specific binding of the antagonist [^3^H]DPCPX binding.

**Fig. 8:**
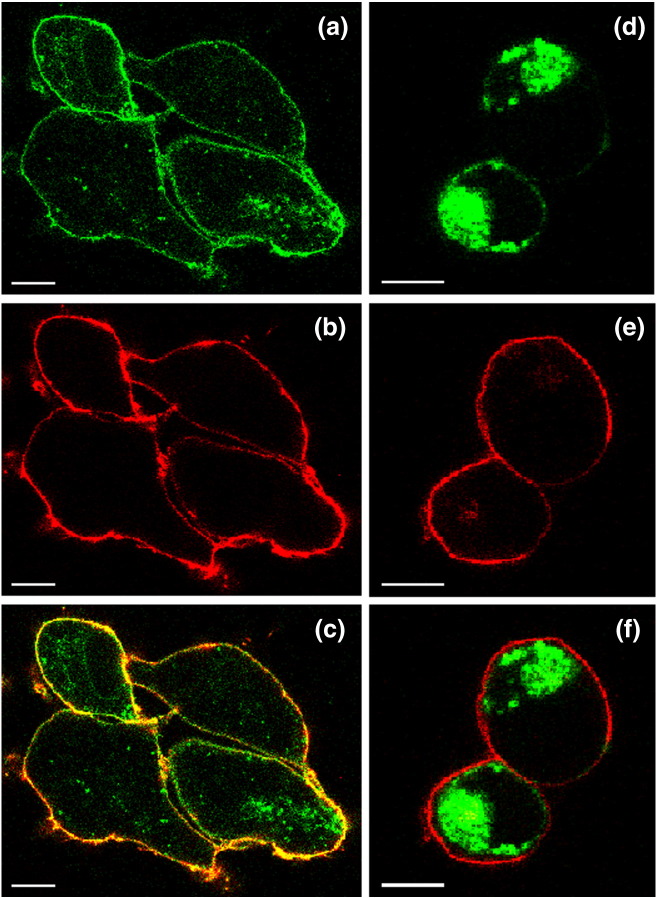
A_1_R-GL26 is misfolded when expressed in mammalian cells. (a–c) Confocal micrographs of the iGnTI^−^(A_1_R-GFP-H_10_) cell line after 24 h of induction with tetracycline. Cells were fixed using paraformaldehyde and the plasma membrane was defined by staining with Alexa Fluor 647-conjugated conA prior to visualisation. Unlabelled iGnTI^−^ parental cells showed no fluorescence (data not shown). The scale bar represents 10 μm. (d–f) Confocal micrographs of the iGnTI^−^(A_1_R-GL26-GFP-H_10_) cell line after 24 h of induction with tetracycline. Cells were fixed using paraformaldehyde and the plasma membrane was defined by staining with Alexa Fluor 647-conjugated conA prior to visualisation. Unlabelled iGnTI^−^ parental cells showed no fluorescence (data not shown). The scale bar represents 10 μm.

**Fig. 9:**
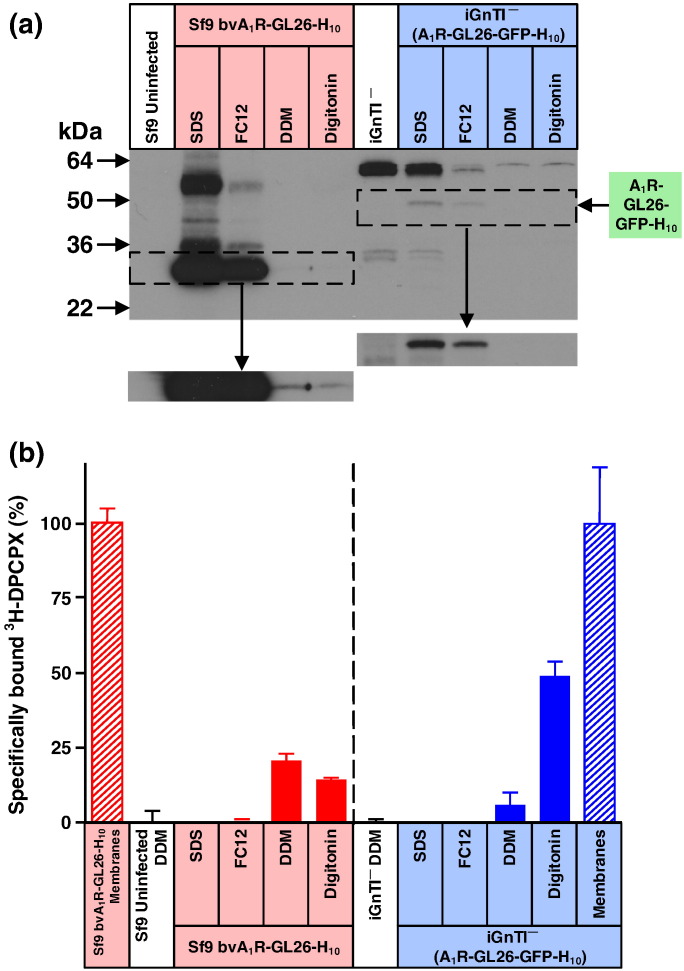
Misfolded A_1_R-GL26 is poorly solubilised either by DDM or digitonin. (a) Western blot of A_1_R solubilised from whole cells using four different detergents (SDS, FC12, DDM or digitonin) and probed with an anti-pentaHis-HRP conjugated antibody. Each lane contains an equal amount of total protein. A_1_R was expressed either in the stable mammalian cell line [iGnTI^−^(A_1_R-GL26-GFP-H_10_)] or by using the recombinant baculovirus bvA_1_R-GL26-H_10_ to infect Sf9 cells. The iGnTI^−^ cell line was induced with 1 μg/ml tetracycline for 24 h and Sf9 cells were infected for 72 h. The Western blot inserts are a 4× longer exposure. (b) The amount of functional detergent-solubilised A_1_R-GL26 was determined by measuring specific binding of the antagonist [^3^H]DPCPX. After the addition of ligand, membranes were solubilised in the detergent indicated and non-bound ligand was separated from  receptor–ligand complex on gel-filtration spin columns and measured by liquid scintillation counting: red-filled bars, A_1_R-GL26 expressed in Sf9 cells; blue-filled bars, A_1_R-GL26 expressed in iGnTI^−^ cells. The amount of A_1_R-GL26 in membranes (non-solubilised) was determined by separation of receptor-bound and free radioligand by filtration through glass fibre plates: red hatched bars, A_1_R-GL26 expressed in Sf9 cells; blue hatched bars, A_1_R-GL26 expressed in iGnTI^−^ cells. For ease of comparison, binding data have been normalised with respect to A_1_R-GL26 in membranes (100%), which is equivalent to 17,400 ± 800 dpm (*n* = 3; 435 fmol per million cells) for baculovirus-infected Sf9 cells and 2000 ± 350 (*n* = 2; 48 fmol per million cells) for iGnTI^−^(A_1_R-GL26-GFP-H_10_) cells. Absolute levels of A_1_R therefore cannot be compared meaningfully between the two expression systems using this bar graph. Binding assays for A_1_R-GL26 contained 150,000 cells. Each data point was determined in duplicate or triplicate from a single experiment and was plotted as mean ± SEM.

In a second example, we compared the expression of the adenosine A_1_ receptor (A_1_R) in both the stable mammalian cell line iGnTI^−^(A_1_R-GFP-H_10_) and the insect cells using the baculovirus expression system (bvA_1_R-H_10_). N-Linked glycosylated sites are found in extracellular regions of A_1_R (extracellular loop 2; Asn148 and Asn159), which produces a glycosylated form in mammalian cells that can be reduced in molecular weight by treatment with PNGase F, whereas the majority of the receptor is unglycosylated in Sf9 cells (Fig. 6). Expression of A_1_R-H_10_ in insect cells gave comparative Western blots analogous to those observed for AT_1_R-H_10_, with SDS and FC12 extracting orders of magnitude more polypeptide from insect cell membranes compared to either DDM or digitonin, consistent with a large excess of misfolded receptor produced in the baculovirus expression system (Fig. 7). In contrast, all the detergents used to solubilise A_1_R-GFP-H_10_ from a stable mammalian cell line were equally efficacious, indicating that there is minimal misfolded receptor in these cells (Fig. 7). The low levels of antagonist binding activity observed for A_1_R is a consequence of the poor stability of this receptor in detergent solutions. A_1_R also provided a nice example of the usefulness of confocal microscopy in defining whether a receptor is likely to be correctly folded. A_1_R-GFP-H_10_ is expressed in the stable cell line predominantly at the cell surface whereas a mutant of A_1_R that contained multiple changes introduced to try and facilitate crystallisation (A_1_R-GL26-GFP-H_10_; see Methods) was expressed predominantly in intracellular membranes (Fig. 8). The confocal data correlated well with the Western blotting data. The misfolded mutant A_1_R-GL26-GFP-H_10_ was only efficiently extracted from mammalian cells with SDS (Fig. 9), whereas the wild-type receptor was extracted equally efficiently using either digitonin or SDS (Fig. 7).

**Fig. 10:**
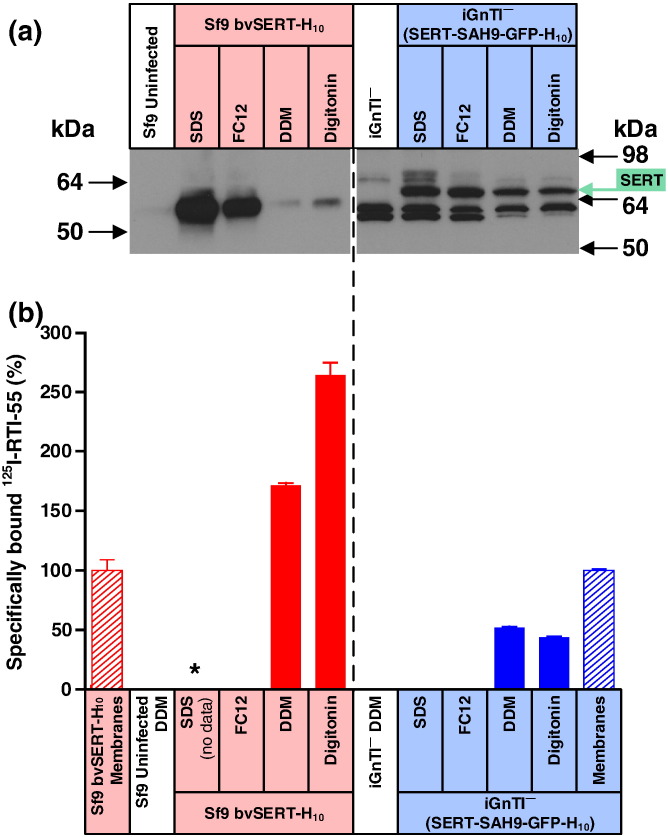
Misfolded SERT produced by the baculovirus expression system is poorly solubilised by either DDM or digitonin. (a) Western blot of SERT solubilised from whole cells using four different detergents (SDS, FC12, DDM or digitonin) and probed with an anti-pentaHis-HRP conjugated antibody. Each lane contains an equal amount of total protein. SERT was expressed either in the stable mammalian cell line iGnTI^−^(SERT-SAH9-GFP-H_10_) or by using the recombinant baculovirus bvSERT-H_10_ to infect Sf9 cells. The iGnTI^−^ cell line was induced with 1 μg/ml tetracycline for 24 h and Sf9 cells were infected for 48 h. The broken line indicates separate blots. (b) The amount of functional detergent-solubilised SERT was determined by measuring specific binding of the ligand [^125^I]RTI-55. After the addition of ligand, membranes were solubilised in the detergent indicated and non-bound ligand was separated from receptor–ligand complex on gel-filtration spin columns and measured by liquid scintillation counting: red-filled bars, SERT expressed in Sf9 cells; blue-filled bars, SERT-SAH9 expressed in iGnTI^−^ cells; *, not determined. The amount of SERT in membranes (non-solubilised) was determined by separation of receptor-bound and free radioligand by filtration through glass fibre plates: red hatched bars, SERT expressed in Sf9 cells; blue hatched bars, SERT-SAH9 expressed in iGnTI^−^ cells. For ease of comparison, binding data have been normalised with respect to SERT in membranes (100%), which is equivalent to 10,200 ± 950 dpm (*n* = 2; 75.7 fmol per million cells) for baculovirus-infected Sf9 cells and 35,400 ± 420 dpm (*n* = 2; 730 fmol per million cells) for iGnTI^−^(Sert-SAH9-GFP-H_10_) cells. Therefore, absolute levels of SERT cannot be compared meaningfully between the two expression systems using this bar graph. Binding assays for SERT contained either 28,000 Sf9 cells or 10,000 iGnTI^−^ cells. Each data point was determined in duplicate or triplicate from a single experiment and was plotted as mean ± SEM.

The final example we tested was the serotonin transporter (SERT). The expression of SERT has been studied intensively [16], [17], [26] and was the first example along with rhodopsin that showed the utility of mammalian cells for the overexpression of functional membrane protein using the tetracycline-inducible HEK293 cell line [27], [28]. Here we demonstrated that the Western blot data show an identical pattern of results with constructs based on wild-type A_1_R and AT_1_R, namely, similar amounts of extractable SERT-SAH9-GFP-H_10_ from the mammalian cell line, regardless of the detergent used, whereas there are orders of magnitude more SDS-extractable SERT-H_10_ in Sf9 cells compared to the amount solubilised by digitonin or DDM (Fig. 10).

### A simplified assay for the detection of misfolded membrane proteins

Analysis of the data in Fig. 1, Fig. 2, Fig. 3, Fig. 4, Fig. 5, Fig. 6, Fig. 7, Fig. 8, Fig. 9, Fig. 10 suggests that the salient conclusions of this paper, that is, that the majority of AT_1_R, A_1_R and SERT constructs expressed in insect cells were misfolded, whereas expression in mammalian cells produced correctly folded protein, could be deduced with a fraction of the work. Comparison of two lanes in each Western blot, namely, SDS-extracted protein and digitonin-extracted protein, is sufficient to draw the relevant conclusions. Importantly, this obviates the need for radioligand binding assays and a stable mammalian cell line for each membrane protein to be studied. Radioligands have been developed for only a small fraction of membrane proteins and not all radioligands are of sufficiently high affinity (100 nM or better) to make them suitable for assays on detergent-solubilised membrane proteins. In addition, construction of stable mammalian cell lines can take many months and sometimes the cell lines grow very poorly due to basal activity of the membrane protein. This was noticeable for the stable A_1_R cell line iGnTI^−^(A_1_R-GFP-H_10_) developed here, which grew very poorly compared to the stable cell line iGnTI^−^(A_1_R-GL26-GFP-H_10_) expressing the inactive A_1_R mutant, despite the use of an inducible promoter.

Using the methodology described in this paper, it would be relatively simple to test 50 or so different membrane protein expression trials in a day. However, if hundreds of samples are to be tested in 96-well plates, then the ultracentrifugation step will become limiting and will need to be replaced using filtration through low-protein-binding 0.2-μm filters. The use of a dot-blot apparatus and semi-quantification of the resulting signals in relation to a known standard would be sufficient to define how much functional membrane protein could be extracted and whether or not extra precautions may be required to remove potential misfolded protein during purification. It would also be possible to measure the fluorescence of a GFP-tagged membrane protein, rather than performing a Western blot, to improve further the high-throughput capabilities of this assay.

## Discussion

A commonly held misconception, particularly amongst investigators new to the membrane protein field, is that if a membrane protein can be expressed into a membrane within a cell and can be extracted with detergent, then that membrane protein is folded authentically. Over the last 30 years, there have been sporadic reports of overexpressed membrane proteins in *E. coli*, yeast or the baculovirus expression system being predominantly misfolded and inactive [2]. The work presented here demonstrates that the baculovirus expression system is particularly prone to producing misfolded membrane proteins, even of apparently uncomplex GPCRs that were expressed over 20 years ago. However, the simple assay proposed here will rapidly demonstrate whether misfolded membrane protein is indeed present. A few words of caution are warranted with regard to the differential solubility assay. Firstly, we have tested the assay on membrane proteins expected to be expressed in the plasma membrane of mammalian cells, which is efficiently solubilised by DDM. This is evident from the similar levels of solubilisation between DDM and SDS of correctly folded membrane proteins in the plasma membrane. Secondly, we are using the assay as a guide rather than as an exact measure for determining the number of molecules of the target membrane protein that are correctly folded compared to the number of molecules that are misfolded.

Knowing that a proportion of an expressed membrane protein is misfolded is important. Many efforts have been made to parallelise expression of membrane proteins to facilitate high-throughput post-genomic approaches to determine rapidly membrane protein structures [3]. Although it has proven possible to do this for bacterial membrane proteins, it has proven harder to replicate these strategies for mammalian membrane proteins, partly because yields of membrane protein suggested from the quantification of polypeptide expressed have not reflected the yield of purified membrane protein. There are two factors that could explain this. Firstly, as described here, most of the membrane protein could be expressed in a misfolded state and therefore cannot be purified in mild detergents. Secondly, membrane proteins are often unstable in detergent and therefore they become inactive and aggregate during solubilisation and purification. The assay described here will define which is the problematic step, thus directing resources to solving the relevant problem. For example, knowing that the majority of membrane protein is misfolded in the baculovirus expression system suggests that using stable inducible mammalian cell lines could improve yields [29]. In the work described here, the AT_1_R expressed in the baculovirus expression system would yield only 0.1 mg/l of functional receptor, whereas the stable clonal cell line iHEK(AT_1_R-GFP-H_10_) would yield 0.5 mg/l. However, the major advantage of using the mammalian expression system is that there is little or no misfolded AT_1_R expressed.

Is the misfolded membrane protein expressed in insect cells a potential problem for downstream purification and crystallisation? Even though DDM is a mild detergent and cannot solubilise misfolded protein as well as SDS or FC12, misfolded AT_1_R is the major component of DDM-solubilised insect cell membranes. In the initial stages of a project, this could be highly misleading, as it would appear that major losses were being incurred on, for example, the first Ni^2 +^-affinity column, when in actual fact, it may be the case that the only protein lost was the misfolded material and that the yields of the correctly folded protein were around 80–90%. In the worst instance, researchers may note that FC12 extracts more of the target protein than DDM and then waste many years trying to purify and crystallise this material, not knowing that the target protein was likely to be totally inactive. Interestingly, the work here shows that β_1_AR is expressed as a mixture of both folded and misfolded receptors, but β_1_AR was purified and crystallised and its structure was determined without knowing this. Two effects may help in reducing the impact of misfolded membrane proteins on crystallisation trials. Firstly, SEC is a frequently used step in protein purification and will effectively remove any misfolded protein. Secondly, misfolded membrane proteins have a tendency to aggregate; thus, this portion may just “disappear”, either through retention on columns by non-specific effects or by being unable to pass through pre-filters that are normally present upstream of columns run on automated protein purification equipment. Thirdly, during crystallisation, any remaining inactive protein will precipitate more readily than the folded protein, hopefully allowing crystals to form later on.

Why are misfolded membrane proteins produced in the baculovirus expression system? Although there are many potential differences between insect cells and mammalian cells that may reduce the efficiency of membrane protein folding (e.g., potential specificity and amounts of molecular chaperones, different lipid composition, etc.), there are two overriding factors that have to be considered. Baculoviruses are lytic viruses, and one of the first effects of the virus is to impair the cells' secretory pathway, which is precisely where membrane proteins are folded. Thus, during the infection cycle, the rate of secretion decreases and it is also observed that post-translational modifications such as N-glycosylation also decrease [30]. In addition, the polyhedrin promoter is one of the strongest known eukaryotic promoters, resulting in the polyhedron mRNA transcript representing over 20% of the cellular polyadeylated RNA [31], [32] and polyhedrin representing over 50% of the total cellular protein upon infection of a wild-type baculovirus [33]. Thus, it is highly likely that production of too much mRNA of a target membrane protein, which could well overwhelm the secretory pathway due to insufficient folding factors, in combination with an impairment in the secretory pathway caused by the baculovirus, combines to facilitate the production of misfolded membrane proteins. It is interesting to note that where careful comparisons have been made with mammalian expression systems that utilise viruses with strong promoters, such as the semiliki forest virus expression system, misfolded and inactive membrane protein has also been observed [19], [34], [35]. Thus, the current successes with the production of authentically folded membrane proteins in mammalian cells for structural studies are all about ensuring that there is a balance between the amount of mRNA produced and the ability of the membrane protein to fold [29]. This will be different for each membrane protein and will have to be optimised empirically on a case-by-case basis. However, the differential solubility assay described here will ensure that expression of only the correctly folded membrane protein will be optimised.

## Materials and Methods

### Materials

All radiolabelled ligands were purchased from PerkinElmer: [^125^I]sar^1^-Ile^8^-angiotensin II ([^125^I]Sar^1^), [^3^H]dihydroalprenolol ([^3^H]DHA), [^3^H]dipropylcyclopentylxanthine ([^3^H]DPCPX) and [^125^I]2β-carbomethoxy-3β-(4-iodophenyl)tropane ([^125^I]RTI-55). The detergents *n*-dodecyl β-d-maltopyranoside (DDM) and fos-choline-12 (FC12) were purchased from Anatrace; SDS was purchased from Sigma and digitonin was purchased from Calbiochem. Anti-penta-histidine antibody conjugated to HRP (anti-pentaHis-HRP) was purchased from Qiagen. A tetracycline-inducible HEK293 cell line, T-Rex™-293 (iHEK), was purchased from Invitrogen. A tetracycline-inducible HEK293S cell line lacking *N*-acetylglucosaminyltransferase I (iGnTI^−^) was kindly provided by Philip J. Reeves (Massachusetts Institute of Technology) [36].

### Methods

#### Constructs

Expression in mammalian cells was performed using derivatives of pcDNA4/TO (Invitrogen). The serotonin transporter cDNA was inserted into the EcoRV/NotI restriction sites in pcDNA4/TO, for expression from the tetracycline-inducible CMV promoter, and then a cassette encoding enhanced GFP, the StrepII tag and a decahistidine (H_10_) tag was inserted after SERT in the NotI/ApaI sites (plasmid pJMA111, kindly provided by J. Andréll, MRC Laboratory of Molecular Biology). The cDNA clone for human angiotensin II type 1 receptor (AT_1_R) was obtained from the Missouri S&T cDNA Resource Center†, amplified by polymerase chain reaction, flanked with EcoRV and NotI sites and cloned into the corresponding sites of pJMA111 to create plasmid pJAP2, which expressed AT_1_R-GFP-H_10_. Additionally, the cDNA for the human adenosine A_1_R (Missouri S&T cDNA Resource Center) was cloned similarly into the EcoRV/NotI sites to create plasmid pJAP34, which expressed A_1_R-GFP-H_10_. In an effort to create a thermostable A_1_R receptor, four mutations that stabilised the adenosine A_2A_ receptor in the active state (L48A, A54L, T65A, Q89A) [37] were transferred to A_1_R (mutations L51A, A57L, L68A, Q92A). In addition, the mutations N148G and N159G were included to remove the putative N-linked glycosylation sites. To remove flexible regions, we truncated the N-terminus between Pro2 and Ile5, truncated the C-terminus at Phe307 and also added the sequence VLRQQEPFKAA to the C-terminus, thus generating A_1_R-GL26. A synthetic cDNA encoding A_1_R-GL26 (Life Technologies) was cloned into the EcoRV/NotI sites in pJMA111 creating pJAP37, which expressed A_1_R-GL26-GFP-H_10_. For generating baculoviruses, AT_1_R was cloned into the BamHI/EcoRI sites of the transfer vector pBacPAK8 (Clonetech), A_1_R was cloned into the XhoI/EcoRI sites and A_1_R-GL26 was cloned into the EcoRI/EagI sites, creating plasmids pJAP15, pJAP44 and pJAP33, respectively. Additionally, AT_1_R was cloned into the BamHI/EcoRI sites in plasmid pAcGP67-B (BD Biosciences) in order to utilise the acidic glycoprotein gp67 signal sequence (LS) preceding the N-terminus of AT_1_R, creating plasmid pJAP16, which expressed AT_1_R-LS-H_10_. All baculovirus sequences were engineered to contain a C-terminal tobacco etch virus cleavage site and H_10_ tag. All constructs were verified by DNA sequencing (Source Biosciences, UK).

#### Transient transfection, generation of stable cell lines and protein expression

Mammalian expression plasmids for the expression of AT_1_R (pJAP2), A_1_R (pJAP34) and A_1_R-GL26 (pJAP37) were amplified in *E. coli* strain DH5α, purified using a Maxi-prep kit (Qiagen) and transiently transfected (GeneJuice, Novagen) into adherent mammalian iHEK cells or iGnTI^−^ cells following the manufacturer's protocol. Cells were grown in Dulbecco's modified Eagle's media supplemented with 10% tetracycline-free foetal bovine serum (Invitrogen) and 5 μg/ml blasticidin (Invitrogen) and incubated at 37 °C in an atmosphere containing 5% CO_2_. Expression of plasmids was induced by addition of 1 μg/ml tetracycline and incubated at 37 °C for 24 h. Stable cell lines were generated by selection with media containing 200 μg/ml Zeocin (Invitrogen). An iGnTI^−^ stable cell line expressing a thermostable mutant of SERT, SERT-SAH9 (J. Andréll and C. Tate, unpublished results; Ref. [38]) and (iGnTI^−^ SERT-SAH9-GFP-H_10_) was kindly provided by J. Andréll. A highly expressing clonal AT_1_R-GFP-H_10_ cell line was selected from a polyclonal cell line using fluorescence-activated cell sorting. After expression, cells were washed twice in phosphate-buffered saline (PBS), counted using the Countess Automated Cell Counter (Invitrogen), pelleted (1200*g* for 5 min) and resuspended at 10 million cells per millilitre in ice-cold cell buffer [50 mM Tris (pH 7.4) and 150 mM NaCl supplemented with Complete EDTA (*e*thylene*d*iamine*t*etraacetic *a*cid)-Free Protease Inhibitor Cocktail (Roche)]. Cell suspensions were flash frozen in liquid nitrogen and stored at − 80 °C.

#### Recombinant baculovirus generation and protein expression

Recombinant baculoviruses that expressed AT_1_R, A_1_R or A_1_R-GL26 were generated using the BaculoGold Baculovirus Expression System according to manufacturer's protocol (BD Bioscience). Viruses were isolated by plaque purification and screened for expression by Western blotting using an anti-pentaHis-HRP antibody. Recombinant baculovirus that expressed SERT with a H_10_ tag at its C-terminus was previously described [16], [26]. Recombinant baculovirus that expressed β_1_AR with a H_10_ tag at its C-terminus [24] was kindly provided by R. Nehme (MRC Laboratory of Molecular Biology) and a thermostable β_1_AR fused to thioredoxin (tsβ_1_AR) was kindly provided by T. Warne (MRC Laboratory of Molecular Biology). Recombinant baculoviruses were passaged twice in Sf9 cells to obtain high titre stocks. Viruses were used to infect Sf9, Sf21 or Hi5 cells for 48 or 72 h as indicated. After protein expression, cells were counted using the Countess Automated Cell Counter (Invitrogen), pelleted (1200*g* for 5 min) and washed twice in PBS, and the cell pellet was resuspended at 10 million cells per millilitre in ice-cold cell buffer [50 mM Tris (pH 7.4) and 150 mM NaCl supplemented with Complete EDTA-Free Protease Inhibitor Cocktail (Roche)]. Cell suspensions were flash frozen in liquid nitrogen and stored at − 80 °C.

#### Western blotting

Cell suspensions were sonicated briefly and the total protein concentration was determined using the Bradford assay [39]. Samples were then solubilised in the detergent indicated [SDS, FC12, DDM or digitonin; all at 1% (w/v) final concentration] at either 4 °C (FC12, DDM, digitonin) or 20 °C (SDS) for 1 h. For blots corresponding to the differential solubility assay, the solubilisate was centrifuged at 280,000*g* for 30 min at 4 °C to remove the insoluble fraction. SDS-loading buffer was added to the supernatant (corresponding to approximately 150,000 cells), and samples were separated on a 4–20% Tris glycine gel and transferred to nitrocellulose using standard techniques. Membranes were probed with anti-pentaHis-HRP at a dilution of 1:1000 and developed using enhanced chemiluminescence (GE Healthcare). Where indicated, 2 μl of PNGase F (New England Biolabs) was added to 15 μl of the supernatant and incubated at 37 °C for 1 h prior to SDS-PAGE to remove N-linked glycosylation.

#### Thermostability assay of detergent-solubilised AT_1_R

The cell suspension containing unpurified AT_1_R was sonicated briefly and diluted into buffer [50 mM Tris (pH 7.4), 5 mM MgCl_2_, 1 mM EDTA, 0.1% (w/v) bovine serum albumin, 150 mM NaCl and 40 μg/ml bacitracin]. [^125^I]Sar^1^ and unlabelled Sar^1^ were added to give final concentrations of 0.5 nM and 100 nM, respectively, and incubated for 1 h at room temperature before chilling on ice and solubilising in 1% DDM (w/v, final concentration). The samples were then heated at varying temperatures for 30 min and the [^125^I]Sar^1^-bound receptor was separated from the free radioligand by gel-filtration spin columns as described previously [40], [41], [42], [43]. Background was determined by adding radioligand to non-transfected parental mammalian cells or uninfected insect cells. Each reaction was performed in triplicate. Results were evaluated by nonlinear regression using GraphPad Prism.

#### Detergent-solubilised and membrane-bound radioligand binding assays

Cell suspensions were sonicated briefly and the total protein concentration was determined using the Bradford assay [39]. Cells were then diluted into buffer [150 mM NaCl and 50 mM Tris (pH 7.4)], incubated with the respective radioligand (1 h, 4 °C) and solubilised in a final concentration of 1% detergent (DDM, FC12, digitonin, SDS) for 1 h at 4 °C. [^3^H]DHA was used at a final concentration of 200 nM and [^3^H]DPCPX was used at a final concentration of 39 nM in 150 mM NaCl and 50 mM Tris (pH 7.4). [^125^I]RTI-55 was used at a concentration of 1 nM in PBS. [^125^I]Sar^1^ was used as per the thermostability assay mentioned above. Bound and free radioligands were separated on gel-filtration spin columns as above.

To determine the amount of SERT, AT_1_R, β_1_AR or A_1_R present in cell membranes, we performed binding assays as mentioned above but without the samples being solubilised with detergent. Separation of receptor-bound and free radioligands was achieved by filtration through a 96-well glass fibre filter plates (Millipore) pre-treated with 0.1% polyethyleneimine [38] except for [^125^I]Sar^1^ where no polyethyleneimine was used. Background for both assays was determined by adding radioligand to non-transfected parental mammalian cells or uninfected insect cells.

#### FSEC and SEC analysed by Western blotting

The void volume (8.16 ml) of the Superdex 200 10/300 (24 ml) (GE healthcare) was determined by running blue dextran through the column and observing where it eluted using *A*_280_. For FSEC, approximately 5 million iHEK(AT_1_R-GFP-H_10_) cells were thawed on ice and sonicated briefly. Cells were incubated at room temperature for 1 h with 40 nM Sar^1^ before chilling on ice and solubilising in 1% DDM (w/v, final concentration). Followed by centrifugation at 280,000*g* for 30 min at 4 °C. The supernatant was then passed through a 0.22-μm filter and injected onto a Superdex 200 10/300 column pre-equilibrated with running buffer [0.03% (w/v) DDM, 50 mM Tris (pH 7.4), 150 mM NaCl and 1 μM Sar^1^]. The fluorescence of eluent was detected by a Hitachi fluorometer (mV) set to an excitation of 488 nm and an emission of 525 nm. Approximately 5 million cells were sonicated, incubated with ligand, solubilised and centrifuged as described above, in order to detect bvAT_1_R-H_10_ produced in Sf9 cells. The eluent was detected by Western blotting as described above and bands corresponding to bvAT_1_R-H_10_ were quantified by densitometry using ImageJ.

#### Fixing and staining cells for analysis by confocal laser-scanning microscopy

Cells were grown on 35-mm glass bottom culture dishes, induced for 24 h under standard conditions and fixed using 2% paraformaldehyde [28]. After washing with PBS, we selectively stained membranes using a solution (10 μg/ml) of concanavalin A (ConA)–Alexa Fluor 647 conjugate (Invitrogen) in PBS for 10 min at room temperature. After washing with PBS, we stored icells n fresh PBS with 0.02% Na azide at 4 °C protected from light. Cells were visualised on a Leica TCS SP8 STED inverted laser-scanning microscope with 63× oil-immersion objective and a 1.4 numerical aperture. The white light laser was set to a wavelength of 488 nm to excite GFP and to 633 nm for Alexa Fluor 647 with the pinhole emission wavelength set to 580 nm.

## Footnotes

† www.cdna.org
